# Prevalence of depression and concomitant risk factors in pediatric patients with rheumatic disease

**DOI:** 10.1186/1546-0096-10-S1-A74

**Published:** 2012-07-13

**Authors:** Stacey E Tarvin, Nicole M Taylor, Christine Raches, Lisa Macharoni

**Affiliations:** 1 Indiana University, Indianapolis, IN, USA; 2 University of Indianapolis, Indianapolis, IN, USA

## Purpose

Chronic disorders, such as rheumatic disease, impact children emotionally, socially and physically. Children who perceive their medical condition interferes with the ability to engage in social, educational and family activities report more depressive symptoms. The purpose of this study is to examine the prevalence of depression in children with rheumatic disease and to identify concomitant risk factors for depression in this population. It was hypothesized that, compared to healthy peers, children with rheumatic diseases may have a higher risk of developing depressive symptoms.

## Methods

Participants were children, 8-17 years old, followed by a pediatric rheumatology practice and identified at a routine follow-up appointment. Children completed the Childhood Depression Inventory (CDI) and parent-child pairs completed a series of standardized measures documenting demographics, symptoms, health-related quality of life, coping mechanisms and psychosocial variables. The primary rheumatologist documented a global assessment of disease severity as well as disease activity scores for children with the diagnosis of juvenile idiopathic arthritis (JIA), juvenile dermatomyositis (JDMS) or systemic lupus erythematosus (SLE). One-way ANOVAs were completed to determine if demographic variables were significantly associated with depression. Pearson product moment correlations were run to determine relationships between the depression, pain, coping, quality of life, family conflict, physical functioning and disease severity. Hierarchal multiple regression analyses were run to determine which medical and psychosocial variables were significant predictors of depression in children with rheumatic disease, while controlling for demographic variables.

## Results

At this time, 96 patients have been screened (78% female) and only data regarding demographics, length of diagnosis and physician documented disease activity has been examined. Five percent of the population was found to have clinically significant symptoms of depression while 15% of the population was found to be “at risk” for symptoms of depression. Seven percent of participants were receiving medication for depression and an additional 7% were involved in therapy at screening. Medication usage significantly correlated with increased CDI scores (increased depressive symptoms) whereas those in therapy had CDI scores comparable to their non-depressed peers. Total CDI score also significantly correlated with female gender and an increased number of children in the home. Neither years since diagnosis, active joint count, total SLEDAI score, JDMS disease activity score nor physician global assessment correlated with increased CDI scores. Additional analyses of psychosocial factors are in progress.

## Conclusion

The prevalence and risk of depression among children with rheumatic diseases is comparable to previously reported data for a healthy population of children. Female gender and number of siblings are associated with increased risk of depressive symptoms. Early data suggests that medications appear to be less efficacious at controlling depressive symptoms than intervention with therapy.

## Disclosure

Stacey E. Tarvin: None; Nicole M. Taylor: None; Christine Raches: None; Lisa Macharoni: None.

